# Safety and short-term outcomes of a modified valvuloplastic esophagogastrostomy versus gastric tube anastomosis after laparoscopy-assisted proximal gastrectomy: a retrospective cohort study

**DOI:** 10.1007/s00464-023-10663-0

**Published:** 2024-01-25

**Authors:** Bailong Li, Yinkui Wang, Zhouqiao Wu, Fei Shan, Shuangxi Li, Yongning Jia, Rulin Miao, Zhemin Li, Kan Xue, Chao Yan, Shen Li, Jiafu Ji, Ziyu Li

**Affiliations:** Key Laboratory of Carcinogenesis and Translational Research (Ministry of Education/Beijing), Gastrointestinal Cancer Center, Peking University Cancer Hospital & Institute, 52 Fucheng Road, Haidian District, Beijing, 100142 China

**Keywords:** Laparoscopic surgery, Proximal gastrectomy, Esophagogastrostomy, Complication, Reflux esophagitis

## Abstract

**Background:**

There is no optimal reconstruction method after proximal gastrectomy. The valvuloplastic esophagogastrostomy can reduce postoperative reflux esophagitis, but it is technically complex with a long operation time. The gastric tube anastomosis is technically simple, but the incidences of reflux esophagitis and anastomotic stricture are higher.

**Methods:**

We have devised a modified valvuloplastic esophagogastrostomy after laparoscopy-assisted proximal gastrectomy (LAPG), the arch-bridge anastomosis. After reviewing our prospectively maintained gastric cancer database, 43 patients who underwent LAPG from November 2021 to April 2023 were included in this cohort study, with 25 patients received the arch-bridge anastomosis and 18 patients received gastric tube anastomosis. The short-term outcomes were compared between the two groups to evaluate the efficacy of the arch-bridge anastomosis. Reporting was consistent with the STROCSS 2021 guideline.

**Results:**

The median operation time was 180 min in the arch-bridge group, significantly shorter than the gastric tube group (*p* = 0.003). In the arch-bridge group, none of the 25 patients experienced anastomotic leakage, while one patient (4%) experienced anastomotic stricture requiring endoscopic balloon dilation. The postoperative length of stay was shorter in the arch-bridge group (9 vs. 11, *p* = 0.034). None of the patients in the arch-bridge group experienced gastroesophageal reflux and used proton pump inhibitor (PPI), while four (22.2%) patients in the gastric tube group used PPI (*p* = 0.025). The incidence of reflux esophagitis (Los Angeles grade B or more severe) by endoscopy was lower in the arch-bridge group (0% vs. 25.0%).

**Conclusion:**

The arch-bridge anastomosis is a safe, time-saving, and feasible reconstruction method. It can reduce postoperative reflux and anastomotic stricture incidences in a selected cohort of patients undergoing laparoscopy-assisted proximal gastrectomy.

**Graphical abstract:**

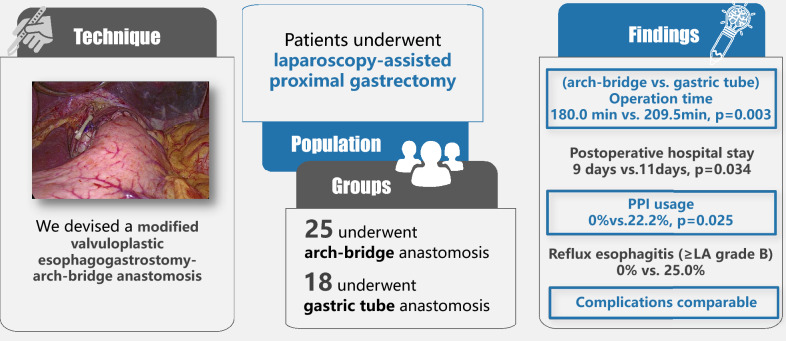

**Supplementary Information:**

The online version contains supplementary material available at 10.1007/s00464-023-10663-0.

Gastric cancer (GC) is one of the most common malignant tumors in the digestive system [1]. Recently, the incidence rates of proximal gastric cancer (PGC) and adenocarcinoma of the esophagogastric junction (AEG) have increased rapidly [2]. Proximal gastrectomy (PG) has received more attention from gastrointestinal surgeons. However, the postoperative complications after PG, such as reflux esophagitis (RE) and anastomotic stricture, severely impair the postoperative quality of life (QoL). To overcome these issues, various methods of digestive tract reconstruction after PG have been reported [3–5], and there is no recognized optimal reconstruction method until now.

The valvuloplastic esophagogastrostomy was developed by Kamikawa in 1998, in which the distal esophagus and the anastomotic site were implanted in the submucosal layer and covered by the seromuscular flap to reduce the postoperative RE [6]. A meta-analysis by Shaibu et al. demonstrated that this reconstruction method could reduce the anastomotic leakage, anastomotic stricture, and residual food than double-tract reconstruction, jejunal interposition, and esophagogastrostomy [7]. It presented an excellent anti-reflux efficacy, with an 8.9% incidence of postoperative RE. Despite these advantages, the completely hand-sewn suturing process under laparoscopy remains the most technically challenging aspect, requiring a long operation time. Many gastrointestinal surgeons still hesitate to perform this operation.

We devised a modified esophagogastric reconstruction method, called arch-bridge anastomosis based on the valvuloplastic esophagogastrostomy [8]. This method can be easily performed with laparoscopic surgery, with a significantly shorter operation time than the conventional valvuloplastic esophagogastrostomy. Meanwhile, it can maintain excellent anti-reflux efficacy. The present study aims to report the surgical outcomes of the arch-bridge anastomosis and compare with the gastric tube anastomosis.

## Materials and methods

### Patients

**Fig. 1:**
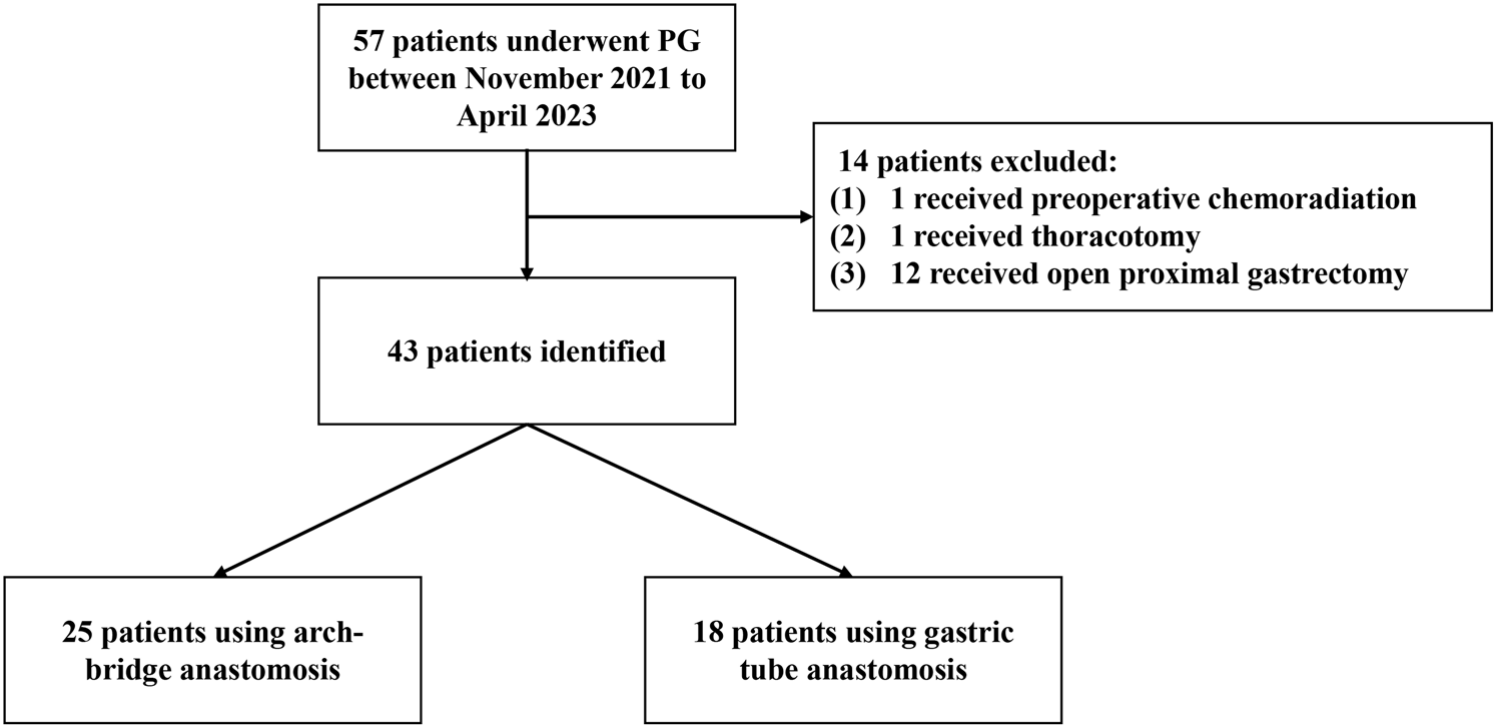
The flowchart of the patients’ selection

This study is a retrospective cohort study to compare the safety and short-term outcomes of patients who underwent arch-bridge anastomosis and gastric tube anastomosis after laparoscopy-assisted proximal gastrectomy (LAPG) in the Department of Gastrointestinal Surgery Ward One, Peking University Cancer Hospital between November 2021 and April 2023. The inclusion criteria were: (1) histologically proven proximal gastric cancer or adenocarcinoma of the esophagogastric junction; (2) patients who received LAPG with arch-bridge anastomosis or gastric tube anastomosis. Exclusion criteria were: (1) preoperative chemoradiation therapy; (2) open proximal gastrectomy; (3) combined with thoracotomy. The flowchart of the patients’ selection is shown in Fig. 1. The reconstruction method, either arch-bridge anastomosis or gastric tube anastomosis, was decided based on each patient’s preference after sufficient description of both procedures. If a patient could not decide on the reconstruction method, the chief, and the surgeon would determine the reconstruction method based on the actual operative circumstances. Preoperative assessment of all patients was performed by a multidisciplinary team. All patients were operated on by an experienced surgical team. The team’s chief surgeon (Ziyu Li) owned over 20 years of clinical practice and experience of over 1000 cases of laparoscopic procedures. This study was approved by the institutional review board of the Peking University Cancer Hospital medical ethics committee (No. 2023YJZ11), and written informed consent was obtained from each patient. This study was registered on the ClinicalTrials with the registration number NCT05829213. The study protocol is provided in the Supplementary File. This study was reported in line with STROCSS criteria [9].

### Surgical and anastomotic technique

All patients enrolled in our study underwent LAPG with D1 + lymph node dissection (No. 1, 2, 3a, 4sa, 4sb, 7, 8a, 9 and 11p) following the Japanese Gastric Cancer Treatment Guideline (version 5) [10]. The patient was placed in the reverse Trendelenburg position with legs open. The surgeon stood at the patient's right side, while one assistant stood on the patient’s left side and one between the patient's legs. The LAPG procedures were performed with five abdominal trocar sites, including one 12 mm trocar below the umbilicus for the camera and additional four trocars for working ports.

### Arch-bridge anastomosis

**Fig. 2:**
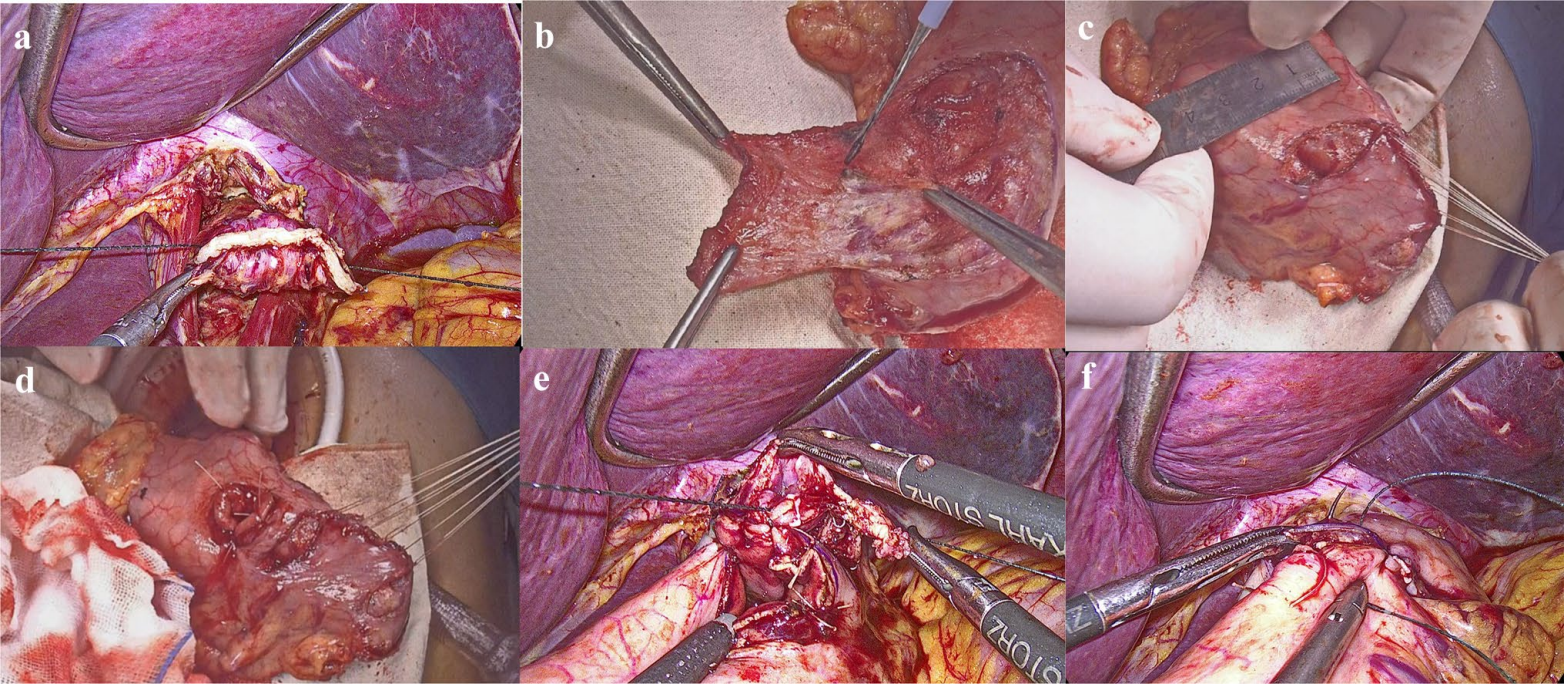
**a** Two barbed threads were sutured on the stapled line of the esophageal stump. **b** A "匚"-shaped single seromuscular flap (3.0 × 4.0 cm) was created on the anterior wall of the remnant stomach. **c** The opening of the single flap was closed by absorbable sutures under direct vision, forming a structure which looked like an arch-bridge. **d** A small hole was opened 1 cm away from the distal edge of the arch-bridge. Four stitches of absorbable sutures were sewed to put gastric mucosal and seromuscular layers together. **e** The anastomosis of the posterior wall was carried out by a continuous suture between the posterior wall of the esophageal stump and the proximal side of the small hole using one barbed thread. **f** Anastomosis of the anterior wall was carried out by continuous suture between the anterior wall of the esophageal stump, the distal side of the small hole and the flap

After the abdominal esophagus had been sufficiently exposed, the esophagus was transected with a laparoscopic linear stapler. After checking the free resection margins, two barbed threads were sutured on the stapled line of the esophageal stump (Fig. 2a). An auxiliary incision was made, and proximal gastrectomy was performed with a linear stapler extracorporeally. Then, a "匚"-shaped single seromuscular flap (3.0 × 4.0 cm) was created on the anterior wall of the remnant stomach, which was 1 cm from the top (Fig. 2b). The opening of the single flap was made towards the lesser curvature. Then the opening of the single flap was closed by 4-0 absorbable sutures under direct vision, forming a structure that looked like an arch-bridge (Fig. 2c). Therefore, this reconstruction method was named arch-bridge anastomosis. This step played an important role in shortening the operation time compared to the intracorporeal sutures of conventional valvuloplastic esophagogastrostomy. After creating the arch-bridge, a small hole (2 cm in diameter) was opened 1 cm away from the distal edge of the arch-bridge. Four stitches of 4-0 absorbable sutures were sewed around the hole to put gastric mucosal and seromuscular layers together (Fig. 2d). This could prevent surgeons from suturing between only the seromuscular layer of the stomach and the esophagus during the following anastomosis.

Pneumoperitoneum was re-established to perform the intracorporeal anastomosis. At this moment, the two pre-sutured barbed threads should be pulled downward to make the esophageal stump through the arch-bridge and reach the anastomotic site- the small hole. Then the stapled line of the esophageal stump was cut using an ultrasound-activated shear. The anastomosis of the posterior wall was carried out by a continuous suture between the posterior wall of the esophageal stump and the proximal side of the small hole using one barbed thread (Fig. 2e). The stapled line of the esophageal stump was cut every 1 cm, and complete the anastomosis with the part that have been cut and the proximal side of the small hole, until complete resection of the stapled line. Anastomosis of the anterior wall was carried out by continuous suture between the anterior wall of the esophageal stump, the distal side of the small hole, and the flap using another barbed thread (Fig. 2f). Finally, the esophagogastrostomy was completed with the anastomotic site and the lower esophagus covered by the arch-bridge. The video of arch-bridge anastomosis is provided in the Supplementary File.

### Gastric tube anastomosis

As for all cases in the gastric tube anastomosis group, esophagogastrostomy was performed by a linear stapler. After the abdominal esophagus had been sufficiently exposed, the esophagus was transected. Then, an auxiliary incision was made, and the stomach was exteriorized through this incision. The gastric body was diagonally divided from the lower portion of the lesser curvature toward the upper part of the greater curvature with a linear stapler to create a gastric tube (20 cm long, 3–4 cm wide). Following this, a small hole was opened 6 cm away from the top of the anterior wall of the remnant stomach as preparation for anastomosis. The pneumoperitoneum was re-established to perform the intracorporeal anastomosis. Before opening the esophageal stump, the esophageal stump was hung by two barbed threads with a spacing of about 1 cm. Then an ultrasound-activated shear made an entry hole on the esophageal stump. After the linear stapler insertion and activation, a side-to-side anastomosis was made between the esophageal stump and the remnant stomach. Then, the common entry hole was closed bidirectionally using the pre-sutured barbed wires. Pyloroplasty was not performed in any of the cases in the present study.

### Data collection and outcome assessment

Demographic characteristics, including age, sex, height, weight, performance score according to the Eastern Cooperative Oncology Group (ECOG), comorbidities, tumor location, and pathological characteristics, were collected from all included patients. Pathological staging was reported according to the 8th edition of the International Union Against Cancer (UICC) TNM classification [11]. The tumor location was determined by preoperative abdominal contrast-enhanced computed tomography (CT).

Surgery-related indices were collected, including operation time, time for anastomosis, estimated blood loss, and number of retrieved lymph nodes. Postoperative recovery-related indices were recorded, including time to first aerofluxus, time to first defecation, time to liquid diet, and postoperative hospital stays. Postoperative complications were defined as conditions that occurred during the hospital stay following surgery and graded using the Clavien-Dindo classification system [12].

### Follow-up

All patients were recommended to receive re-examinations in outpatient clinic every 3 months in the first 2 years after surgery, and every 6 months in the next 3 years. The routine follow-up included physical examinations, laboratory blood tests, and computed tomography or abdominal ultrasonography. If patients developed reflux symptoms during the follow-up, such as heartburn and acid regurgitation, proton pump inhibitor (PPI) would be prescribed by physicians. Endoscopy was performed once a year. Reflux esophagitis was evaluated by endoscopy 12 months after surgery and classified by Los Angeles classification [13].

### Statistical analysis

Continuous variables were displayed as mean ± standard deviation, and categorical variables were displayed as frequency (percentage). The results were presented as the medians (interquartile ranges, IQRs) for high-skew data. Independent t-tests, Mann–Whitney *U* tests, Chi-square tests, and Fisher's exact tests were used to determine differences between the two groups' baseline data and surgical outcomes. All reported *p* values were two-sided, and *p* value < 0.05 was considered significant. SPSS, Version 26.0 (IBM Corporation, Armonk, NY, USA) performed all statistical analysis analyses.

## Results

### Demographic and clinicopathological characteristics

A total of 43 patients were included in the study, including 25 patients receiving LAPG with arch-bridge anastomosis and 18 patients receiving LAPG with gastric tube anastomosis. The demographic and clinicopathological characteristics of the two groups, including age, sex, ECOG performance status, BMI, tumor location, tumor size, previous abdominal history, histological type, and pathological stage, were comparable. The detailed characteristics of the two groups are shown in Table 1.

**Table 1:** Demographic and clinicopathological characteristics of the two groups

Clinical parameters	Arch-bridge group (*n* = 25)	Gastric tube group (*n* = 18)	*p* value
Age (years)	62 (55,70)	60 (55,67)	0.444
Sex			1000
Male	21 (84.0)	15 (83.3)	
Female	4 (16.0)	3 (16.7)	
ECOG PS			0.740
0	14 (56.0)	11 (61.1)	
1	11 (44.0)	7 (38.9)	
BMI (kg/m^2^)	25.4 ± 3.1	24.5 ± 3.5	0.399
Tumor location			0.229
EGJ	12 (44.0)	12 (66.7)	
U	13 (56.0)	6 (33.3)	
Tumor size (cm)	2.0 (1.8,3.0)	2.5 (1.7,4.0)	0.610
Previous abdominal surgery			0.683
Yes	3 (12.0)	3 (16.7)	
No	22 (88.0)	15 (83.3)	
Histological type			0.683
Adenocarcinoma	22 (88.0)	15 (83.3)	
SRC	3 (12.0)	3 (16.7)	
Pathological stage			0.496
IA	10 (40.0)	7 (38.9)	
IB	9 (36.0)	1 (5.6)	
IIA	1 (4.0)	6 (33.3)	
IIB	0 (0.0)	2 (11.1)	
IIIA	5 (20.0)	2 (11.1)	

*ECOG PS* Eastern cooperation Oncology Group performance status, *BMI* body mass index, *EGJ* esophagogastric junction, *U* upper third, *SRC* signet-ring cell carcinoma

### Surgical outcomes and postoperative recovery parameters

The operation time was shorter in the arch-bridge anastomosis group than in the gastric tube anastomosis group [median (IQR) 180 (171.5,201) minutes vs. 209.5 (191.8,217) minutes, *p* = 0.003]. There was no significant difference in time for anastomosis laparoscopically between the two groups [median (IQR) 22 (18,25.5) minutes vs. 19 (18,35) minutes, *p* = 0.540]. The mean time for creating the arch-bridge extracorporeally was 14 min. No patient needed combined organ resection among the two groups. No significant difference was found in estimated blood loss between the two groups (*p* = 0.178). A comparable number of lymph nodes (LNs) were retrieved in both groups, with the median (IQR) number of LNs per patient being 29 (22,33) in the arch-bridge group and 24 (18,36) in the gastric tube group (*p* = 0.739).

No cases of conversion to open surgery were observed in both groups. The postoperative complication rates were 16.0% in the arch-bridge group and 33.3% in the gastric tube group, with no significant difference (*p* = 0.275). In the arch-bridge anastomosis group, one patient developed vomiting after taking a semi-liquid diet 1 month after surgery, then he was diagnosed with anastomotic stricture by endoscopy and was successfully treated with endoscopic balloon dilatation. Respiratory infection was observed in two patients, and pulmonary embolism was observed in one patient. However, no postoperative complications were observed in the arch-bridge group, including anastomotic leakage and anastomotic bleeding. In the gastric tube anastomosis group, anastomotic leakage was observed in one patient, and intra-abdominal infection was observed in two patients. All these patients were recovered through conservative treatment. There were no cases of mortality and reoperation in both groups.

There was no significant difference between the two groups in time to first flatus (*p* = 0.108), time to first defecation (*p* = 0.234), and time to first liquid diet (*p* = 0.585). The postoperative length of stay was shorter in the arch-bridge anastomosis group than in the gastric tube anastomosis group [median (IQR) 9 (7,10) days vs. 11 (8,12) days, *p* = 0.034]. None of the arch-bridge anastomosis group patients complained of postoperative gastroesophageal reflux and used PPI. On the other hand, a PPI was prescribed for four (22.2%) patients with symptoms of heartburn or acid regurgitation in the gastric tube anastomosis group (*p* = 0.025). The details of surgical outcomes and postoperative recovery parameters are provided in Table 2.

**Table 2:** Comparison of surgical outcomes and postoperative parameters between arch-bridge anastomosis group and gastric tube anastomosis group

Outcomes	Arch-bridge group (*n* = 25)	Gastric tube group (*n* = 18)	*p* value
Operation time (min)	180 (171.5,201)	209.5 (191.8,217)	0.003
Time for anastomosis (min)	22 (18,25.5)	19 (18,35)	0.540
Estimated blood loss (ml)	100 (50,100)	100 (100,113)	0.178
The number of retrieved LNs	29 (22,33)	24 (18,36)	0.739
Postoperative complications	4 (16.0%)	6 (33.3%)	0.275
Anastomotic leakage	0 (0%)	1 (5.6%)	0.419
Anastomotic stenosis	1 (4.0%)	0 (0%)	1.000
Anastomotic bleeding	0 (0%)	0 (0%)	1.000
Intra-abdominal infection	0 (0%)	1 (5.6%)	0.419
Respiratory infection	2 (8.0%)	1 (5.6%)	1.000
Pulmonary embolism	1 (4.0%)	0 (0%)	1.000
Other systems infection	0 (0%)	3 (16.7%)	0.066
Mortality	0 (0%)	0 (0%)	1.000
Time to first flatus (days)	4 (3,5)	3 (3,4)	0.108
Time to first defecation (days)	5 (4,5)	5 (3,5)	0.234
Time to first liquid diet (days)	4 (3,5)	5 (3,6)	0.585
Length of stay (days)	9 (7,10)	11 (8,12)	0.034
Use of PPI	0 (0%)	4 (22.2%)	0.025

*LNs* lymph nodes, *PPI* proton pump inhibitor

**Fig. 3:**
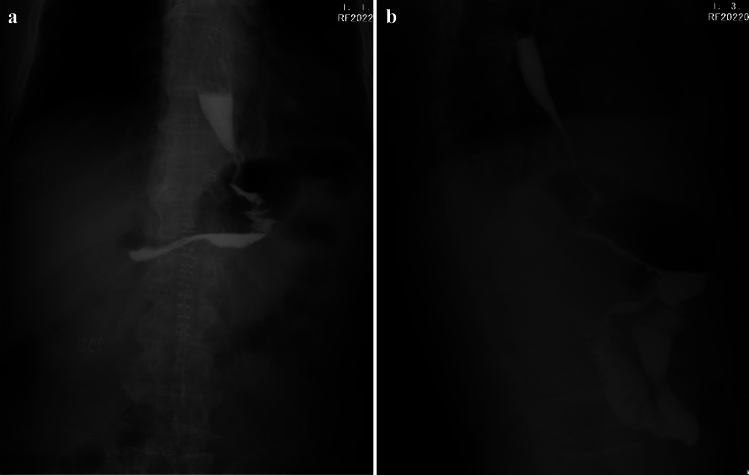
Upper gastrointestinal radiology 7 days after LAPG with arch-bridge anastomosis. **a** Anteroposterior film **b** Lateral film

**Fig. 4:**
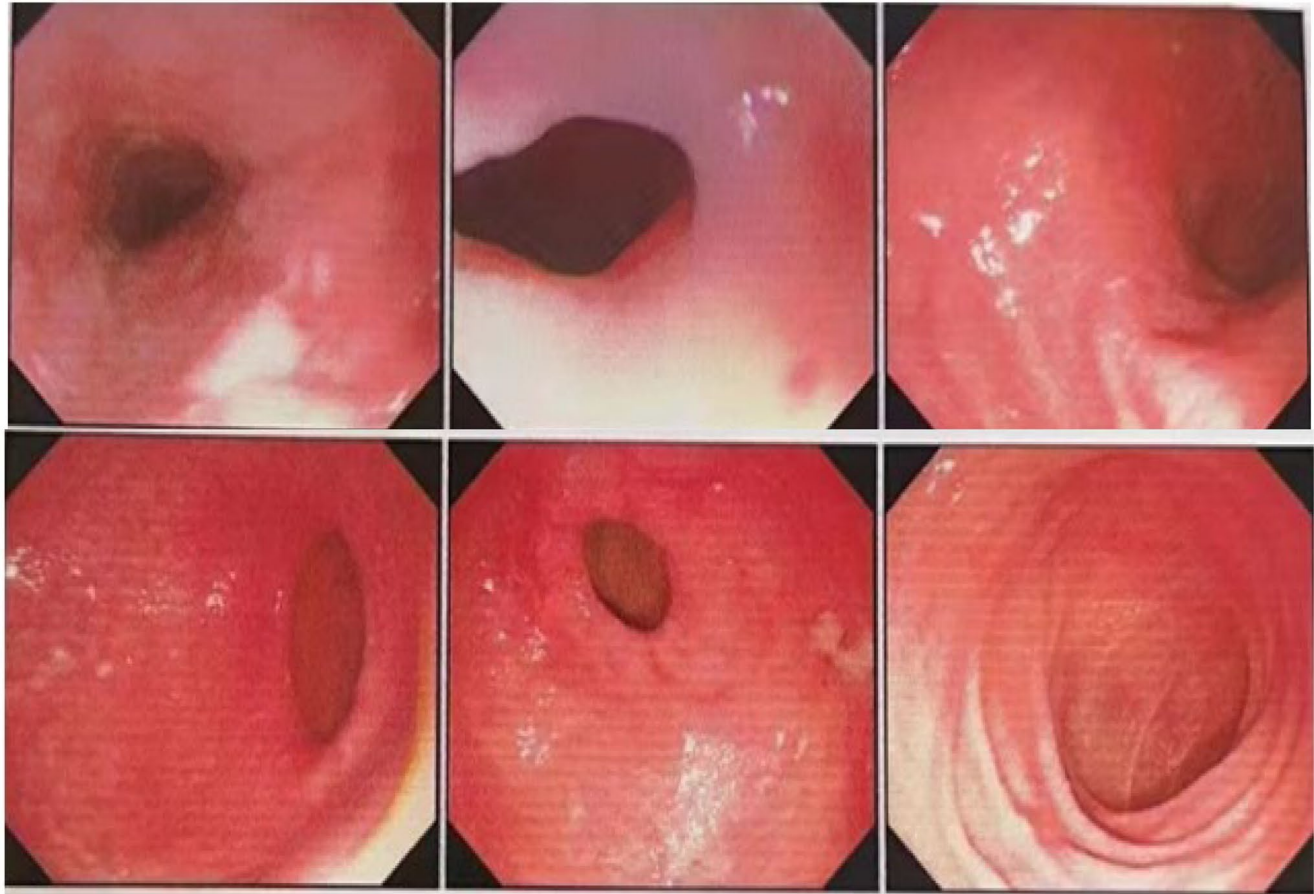
Endoscopic findings 12 months after LAPG with arch-bridge anastomosis

Figure 3 shows the upper gastrointestinal radiology seven days after LAPG with arch-bridge anastomosis. The flow of iodine agent from the esophagus to the remnant stomach was good, and no reflux or extravasation of contrast agent was observed. The median follow-up time was 15.3 months. Thirteen patients in arch-bridge anastomosis group and eight patients in gastric tube group underwent endoscopic examination 12 months after surgery. Among these patients, reflux esophagitis (Los Angeles grade B or more severe) was present in 0% of the arch-bridge group patients and 25.0% in the gastric tube group (Table 3). Figure 4 shows endoscopic findings, and the gastroscopy was inserted smoothly through the anastomosis, without reflux esophagitis or stricture.

**Table 3:** Endoscopic findings 12 months after proximal gastrectomy

Reflux esophagitis (Los Angeles classification)	Arch-bridge group (*n* = 13)	Gastric tube group (*n* = 8)
Grade A	1 (7.7%)	1 (12.5%)
Grade B	0 (0%)	0 (0%)
Grade C	0 (0%)	2 (25.0%)
Grade D	0 (0%)	0 (0%)
Grade B/C/D	0 (0%)	2 (25.0%)

## Discussion

Compared to total gastrectomy (TG), PG can preserve the remnant stomach's physiologic function, leading to better nutritional status after surgery [14]. In the Japanese Gastric Cancer Treatment Guideline 2021 (6th edition), PG is suggested for cT1N0 PGC and esophagogastric junctional cancer to preserve more than half of the distal stomach [15]. Additionally, Yura et al. reported that the metastatic rates at #4d, #5, #6, and #12a lymph nodes were very low in T2/T3 PGC (0.99%, 0, 0, 0.006%, respectively), and PG would be the choice and oncologically safe for these patients [16]. Even so, many surgeons tend to select TG for patients diagnosed with PGC or AEG because the postoperative complications after PG, such as reflux esophagitis, anastomotic leakage, and anastomotic stricture, can lead to a severe decline in patients' QoL.

The reconstruction methods after PG include esophagogastric anastomosis and esophagojujenal anastomosis. Esophagogastrostomy is a conventional reconstruction method with technical simplicity and safety advantages. However, it is reported that over one-fourth of patients developed RE after esophagogastrostomy [7]. This proportion could decrease after a modified esophagogastrostomy, such as SOFY or fundoplication [17, 18]. However, in clinical practice, these procedures require the preservation of the abdominal esophagus and a large remnant stomach, which limits surgery applications.

Valvuloplastic esophagogastrostomy is a modified esophagogastric anastomosis, which increases the pressure of the lower esophagus to prevent postoperative RE [5]. In a multicenter retrospective study, the incidence of RE after valvuloplastic esophagogastrostomy was 10.6%, and that of Los Angeles Grade B or higher by endoscopy was 6.0% [19]. Compared to conventional esophagogastrostomy, it can obtain a satisfactory anti-reflux efficacy. However, in previous studies, the mean operation time for valvuloplastic esophagogastrostomy was long, ranging from 298 to 420 min [5, 20–22]. In addition, it was reported that the incidence of anastomotic stricture was from 5.5 to 29.1% [19, 23]. To overcome these disadvantages, various modified esophagogastrostomy techniques have been explored [24, 25]. In the present study, our team devised this arch-bridge anastomosis, aiming to simplify the surgical procedures and shorten the operation time while maintaining anti-reflux's efficacy. Moreover, to our best knowledge, this is the first study to compare the surgical outcomes between valvuloplastic esophagogastrostomy and gastric tube anastomosis.

In the present study, the proton pump inhibitor usage was significantly fewer in the arch-bridge anastomosis group than the gastric tube anastomosis group. PPI was prescribed for patients who suffered from reflux symptoms. No patients in arch-bridge anastomosis group used PPI postoperatively. However, four patients in gastric tube anastomosis group required long-term use of PPI to relieve reflux symptoms. The results showed that the arch-bridge anastomosis was better than the gastric tube anastomosis in avoiding the occurrence of postoperative reflux. Additionally, two patients in the gastric tube anastomosis group were confirmed to have grade C reflux esophagitis on endoscopy 12 months after surgery. In contrast, although one patient was diagnosed with grade A reflux esophagitis, the remaining 12 patients in arch-bridge anastomosis group showed no evidence of reflux esophagitis on endoscopy. These results indicated that the arch-bridge anastomosis could obtain a better anti-reflux efficacy. Previous studies have reported that the incidence of reflux esophagitis (≥ grade B) following valvuloplastic esophagogastrostomy was 0–6% which was lower than the gastric tube anastomosis [20]. Our results supported the idea that the valvuloplastic technique had a favorable anti-reflux efficacy than the gastric tube anastomosis.

Notably, in our study the median total operation time was 180 min for the arch-bridge anastomosis, which was significantly shorter than that of the gastric tube anastomosis (209.5 min, *p* = 0.003). The time for anastomosis laparoscopically was comparable between the two groups. Although both groups are esophagogastric anastomoses, our results showed that arch-bridge anastomosis was more time-saving than the gastric tube anastomosis. The reason why the arch-bridge anastomosis was more time-saving were as follows: First, we closed the single flap by absorbable sutures under direct vision to form the arch-bridge. The mean time for creating the arch-bridge extracorporeally was 14 min in the present study. This step could markedly reduce the difficulty of suture laparoscopically, because the laparoscopic esophagogastric anastomosis was performed on the caudal side of the arch-bridge, which was located in a lower position and easy to perform. Second, we used the modified overlap method using knotless barbed sutures (MOBS) which was developed in totally laparoscopic gastrectomy [26]. We pulled downward the pre-sutured barbed threads to make the esophageal stump easily through the arch-bridge. On the other hand, the barbed threads could be used for hand-sewn of esophagogastrostomy, and play the role of traction of the esophagus during the anastomosis.

In addition, there was no anastomotic leakage after arch-bridge anastomosis in the present study. This result was consistent with previous studies, because the seromuscular flap covered the anastomotic site. The postoperative complication rates were 16.0% in the arch-bridge anastomosis, suggesting that this reconstruction method was technically safe. On the other hand, anastomotic stricture is a postoperative complication that requires careful attention in this reconstruction method. Only one patient (4%) developed anastomotic stricture after arch-bridge anastomosis and was successfully treated with endoscopic balloon dilation. The incidence of anastomotic stricture in arch-bridge anastomosis was lower than that of the conventional valvuloplastic esophagogastrostomy [19, 23]. We considered that closure of the seromuscular flap under the direct version might improve the suture quality and lead to a lower incidence of anastomotic stricture. Moreover, the postoperative hospital stay was found significant shorter in the arch-bridge anastomosis group. As a new technique on the potential learning curve, clinicians might prolong the time for removing the stomach tube or first liquid diet for surgical safety’s sake. However, the postoperative hospital stay was still shorter, indicating that the patients’ recovery was well in the arch-bridge anastomosis group.

There were several limitations in our study. First, this is a retrospective single-center cohort study with a small sample size. The selection bias was inevitable. Second, owing to the short follow-up time, not all patients in the present study completed postoperative endoscopic examination. Therefore, we performed subgroup analyses in patients who receiving endoscopy and not receiving endoscopy in both groups (Supplementary table). The subgroup analyses found that there was no significant baseline and short-term outcomes difference between patients who receiving endoscopy or not receiving endoscopy. Third, the postoperative long-term QoL should be comprehensively evaluated by symptoms, living status, and QoL, and survey questionnaires needed to be employed, such as the Postgastrectomy Symptom Assessment Scale 45 (PGSAS-45) [27]. Despite these limitations, the present study provides the short-term comparative outcomes of arch-bridge anastomosis and gastric tube anastomosis after LAPG. The prospective study to assess the long-term QoL of patients who underwent arch-bridge anastomosis is being conducted in our center. Multicenter randomized controlled trials are needed to provide evidence of higher level.

In summary, we have devised arch-bridge anastomosis, a modified valvuloplastic esophagogastrostomy after laparoscopy-assisted proximal gastrectomy. This reconstruction method is safe, time-saving and feasible. It may reduce postoperative reflux and anastomotic stricture incidences in a selected cohort of patients undergoing laparoscopy-assisted proximal gastrectomy.

### Supplementary Information

Below is the link to the electronic supplementary material.

Supplementary file1 (DOCX 42 kb)

Supplementary file2 (DOCX 33 kb)

Supplementary file3 (DOCX 20 kb)

Supplementary file4 (MP4 470233 kb)
