# Supplementary material for: Safety and short-term outcomes of a modified valvuloplastic esophagogastrostomy versus gastric tube anastomosis after laparoscopy-assisted proximal gastrectomy: a retrospective cohort study
Source: Surg Endosc. 2024 Jan 25;38(3):1523–32. doi: 10.1007/s00464-023-10663-0 (PMC10881610; doi:10.1007/s00464-023-10663-0)
Supplement: Supplementary file 1 — Supplementary file1 (DOCX 42 kb) [file 464_2023_10663_MOESM1_ESM.docx]

**Study protocol**

**Safety and short-term outcomes of** **a modified valvuloplastic esophagogastrostomy versus gastric tube anastomosis after laparoscopy-assisted proximal gastrectomy: a retrospective cohort study**

Project source: Investigator initiated trial

Principal investigator: Ziyu Li

Department: Department of Gastrointestinal Surgery Ward One, Peking University Cancer Hospital

Tel: +86 010-8819-6606

Execution time: From November 2021 to April 2023

**Introduction**

Gastric cancer is one of the most common malignant tumors in digestive system. According to the data of GLOBOCAN 2020, gastric cancer had the fifth-highest morbidity rate and the fourth-highest mortality rate [1]. East Asia is a high-risk region for gastric cancer. In recent years, the incidence of adenocarcinoma of esophagogastric junction (AEG) and proximal gastric cancer (PGC) have been increasing[2]. A registration study from West China showed that the proportion of AEG increased from 22.3% to 35.7% between 1988 and 2012[3]. Total gastrectomy (TG) is common for AEG and PGC in clinical practice. However, the nutritional deficiency caused by total gastrectomy is inevitable. Proximal gastrectomy (PG) preserves part of the stomach's function and has attracted more attention.

However, the postoperative complications after PG, such as reflux esophagitis and anastomotic stricture, severely impair the postoperative quality of life (QoL). To overcome these issues, various methods of digestive tract reconstruction after PG have been reported, such as double-tract reconstruction, gastric tube reconstruction and jejunal interposition[4, 5]. However, there is a lack of high-level evidence to compare the safety and postoperative QoL of reconstruction methods after PG. In the "Chinese Expert Consensus on Proximal Gastrectomy and Digestive Tract Reconstruction (2020 Edition)", gastric tube anastomosis is a recommended reconstruction method after PG and widely applied in clinical practice[6]. The previous study reported that the incidence of reflux esophagitis (RE) after gastric tube anastomosis was 14%[7].

Valvuloplastic esophagogastrostomy is a modified esophagogastric anastomosis, which increases the pressure of the lower esophagus to prevent postoperative RE. However, in previous studies, the mean operation time for valvuloplastic esophagogastrostomy was long, ranging from 298 to 420 minutes[8]. We devised a modified valvuloplastic esophagogastric reconstruction method after laparoscopy-assisted proximal gastrectomy (LAPG), called arch-bridge anastomosis[9]. The surgery is simplified and the operation time is significantly shortened compared to the conventional valvuloplastic esophagogastric anastomosis. Patients had no reflux symptoms such as acid regurgitation and heartburn in the first ten cases.

The present study aims to compare the safety and short-term outcomes of the arch-bridge anastomosis and the gastric tube anastomosis, and to further evaluate the efficacy of arch-bridge anastomosis.

**Methods**

**Study design**

This study is a single-center, retrospective cohort study. The participants consist of all adult patients underwent arch-bridge anastomosis or the gastric tube anastomosis in the Department of Gastrointestinal Surgery Ward One, Peking University Cancer Hospital from November 2021 to April 2023.

**Study population**

Patients who underwent laparoscopy-assisted proximal gastrectomy with arch-bridge anastomosis or gastric tube anastomosis in the Department of Gastrointestinal Surgery Ward One, Peking University Cancer Hospital are included.

The inclusion criteria are as follows: (1) age≥18; (2) Eastern Cooperative Oncology Group (ECOG) performance status≤2; (3) Histologically proven adenocarcinoma of esophagogastric junction or proximal gastric cancer; (4) Patients who underwent LAPG with arch-bridge anastomosis or gastric tube anastomosis; (5) No evidence of distant metastasis, including positive peritoneal cytology; (6) Informed consent was obtained.

The exclusion criteria are as follows: (1) ECOG performance status＞2; (2) Clinically diagnosed distant metastasis; (3) Remnant gastric cancer, recurrent gastric cancer, or multi-primary cancers; (4) Preoperative chemoradiation therapy; (5) Patients who underwent open proximal gastrectomy; (6) Combined with thoracotomy; (7) Underwent emergency surgery.

**Sample size**

This study is a retrospective cohort study, and all consecutive patients who met the inclusion criteria during the study period are included. According to the surgical volume of LAPG in Department of Gastrointestinal Surgery Ward One in Peking University Cancer Hospital, the arch-bridge anastomosis and gastric tube anastomosis are estimated at 15 cases per year each. The study is expected to include about 20 patients in arch-bridge anastomosis group and the gastric tube anastomosis group each.

**Operator**

The operations were completed by an experienced surgical team, and the chief surgeon met all of the following requirements:

(1) A senior title; (2) Expertise in proximal gastrectomy and digestive tract reconstruction and own experience of over 1000 cases of laparoscopic procedures.

**Surgical techniques**

**Arch-bridge anastomosis**

(1) After the abdominal esophagus is sufficiently exposed, the esophagus is transected with a linear stapler. Two barbed threads are sutured on the stapled line of the esophageal stump.

(2) An auxiliary incision is made, and proximal gastrectomy is performed with a linear stapler extracorporeally.

(3) A "匚"-shaped single seromuscular flap (3.0◊4.0cm) is created on the anterior wall of the remnant stomach, which is 1cm from the top. The opening of the single flap is made towards the lesser curvature. The opening of the single flap is closed by absorbable sutures, forming a structure that looked like an arch-bridge. After creating the arch-bridge, a small hole (2cm in diameter) is opened 1cm away from the distal edge of the arch-bridge. Four stitches of absorbable sutures are sewed around the hole to put gastric mucosal and seromuscular layers together

(4) Pneumoperitoneum is re-established to perform the intracorporeal anastomosis. The two pre-sutured barbed threads should be pulled downward to make the esophageal stump through the arch-bridge and reach the anastomotic site- the small hole.

(5) The stapled line of the esophageal stump is cut using an ultrasound-activated shear. The anastomosis of the posterior wall is carried out by a continuous suture between the posterior wall of the esophageal stump and the proximal side of the small hole using one barbed thread. Anastomosis of the anterior wall is carried out by continuous suture between the anterior wall of the esophageal stump, the distal side of the small hole, and the flap using another barbed thread. Finally, the esophagogastrostomy is completed with the anastomotic site and the lower esophagus covered by the arch-bridge.

**Gastric tube anastomosis**

(1) Lymph node dissection is performed according to the Japanese Gastric Cancer Treatment Guideline[10]. After the abdominal esophagus is sufficiently exposed, the esophagus is transected.

(2) Auxiliary incision is made, and the stomach is exteriorized through this incision. The gastric body is diagonally divided from the lower portion of the lesser curvature toward the upper part of the greater curvature with a linear stapler to create a gastric tube (20cm long, 3-4cm wide). Following this, a small hole is opened 6cm away from the top of the anterior wall of the remnant stomach as preparation for anastomosis.

(3) The pneumoperitoneum is re-established to perform the intracorporeal anastomosis. Before opening the esophageal stump, the esophageal stump is hung by two barbed threads with a spacing of about 1cm. Then an ultrasound-activated shear makes an entry hole on the esophageal stump. After the linear stapler insertion and activation, a side-to-side anastomosis is made between the esophageal stump and the remnant stomach. The common entry hole is closed bidirectionally using the pre-sutured barbed wires.

**Data collection**

**Inpatient medical record system**

Clinical information is collected via the inpatient electronic medical record system and stored using Microsoft Excel 2019. The following variables are extracted: (1) Demographic data, including age, sex, height, weight, ECOG performance score and comorbidities. (2) Tumor characteristics, including tumor location, diameter of tumor, histological type and pathological stage. Pathological stage is reported according to the 8th edition of the International Union Against Cancer (UICC) TNM classification. (2) Surgery-related indices, including estimated blood loss, and number of retrieved lymph nodes. (3) Postoperative recovery-related indices, including time to first flatus, time to first defecation, time to liquid diet, and postoperative hospital stays. (4) Surgical safety indices, including postoperative complications, reoperation and perioperative mortality. Postoperative complications are graded using the Clavien-Dindo classification system.

**Video database**

The whole process of surgery is recorded using camera routinely in our center. By searching the video database, we can find the surgery videos of arch-bridge anastomosis and gastric tube anastomosis. The total operation time, and the time of laparoscopic esophagogastric anastomosis of two reconstruction methods are recorded in details. In the arch-bridge anastomosis group, the time of creating the "arch bridge" extracorporeally needs to be recorded.

**Outpatient medical record system**

All patients in our center receive re-examinations in outpatient clinic every 3 months in the first 2 years after surgery, and every 6 months in the next 3 years. Reflux esophagitis is evaluated by endoscopy 12 months after surgery and classified by Los Angeles classification. The gastroscopy findings 12 months after surgery are extracted by searching outpatient medical record system. The incidence and grading of reflux esophagitis are recorded. Additionally, we extract the patients’ reflux symptoms and proton pump inhibitor usage from outpatient system.

**Outcome assessment**

(1) Primary outcome

Surgical safety: Complication incidence rates during the hospital stay after laparoscopy-assisted proximal gastrectomy with arch-bridge anastomosis or gastric tube anastomosis. Postoperative complications will be graded using the Clavien-Dindo classification system[11]. Postoperative death: all-cause death that occurs from the beginning of surgery to 30 days after surgery. Unplanned reoperation: unplanned reoperation that occurs from the beginning of surgery to 30 days after surgery.

(2) Secondary outcomes:

1) Surgical-related indexes: The total operation time, the time of esophagogastric anastomosis, the estimated blood loss, and the number of retrieved lymph nodes are compared between the two reconstruction methods.

2) Postoperative recovery: The first flatus time, first defecation time, first liquid diet time, and the length of postoperative stay are compared between the two reconstruction methods.

3) Reflux esophagitis: The incidence of reflux esophagitis is compared between the two reconstruction methods. Reflux esophagitis is evaluated by endoscopy 12 months after surgery and classified by Los Angeles classification. The gastroesophageal reflux symptoms and proton pump inhibitors usage in the two reconstruction methods are compared.

**Statistical analysis**

The continuous variables such as age and BMI are described as the mean ± standard deviation if the Kolmogorov–Smirnov test is consistent with a normal distribution. Otherwise, the median (interquartile ranges, IQRs) is used. For the classified variables such as gender and tumor location, we describe the number of cases and percentage. Differences in variables among groups are tested using Student’s t-test, chi-square test, or Fisher’s exact test. Non-parametric test (Mann–Whitney test) will be used for variables with skewed distributions. Bilateral p<0.05 is considered statistically significant. SPSS, Version 26.0 (IBM Corporation, Armonk, NY, USA) performs all statistical analysis analyses.

**Ethics and dissemination**

This study protocol was registered on the ClinicalTrials with the registration number NCT05829213. Additionally, this study was approved by the institutional review board of the Peking University Cancer Hospital medical ethics committee (No. 2023YJZ11). The findings of this study will be published in a peer-reviewed medical journal.

**References**

1. Bray F, Ferlay J, Soerjomataram I, Siegel RL, Torre LA, Jemal A (2018) Global cancer statistics 2018: GLOBOCAN estimates of incidence and mortality worldwide for 36 cancers in 185 countries. CA: a cancer journal for clinicians.68(6):394-424.

2. Buas MF, Vaughan TL (2013) Epidemiology and risk factors for gastroesophageal junction tumors: understanding the rising incidence of this disease. Seminars in radiation oncology.23(1):3-9.

3. Liu K, Yang K, Zhang W, Chen X, Chen X, Zhang B, Chen Z, Chen J, Zhao Y, Zhou Z, Chen L, Hu J (2016) Changes of Esophagogastric Junctional Adenocarcinoma and Gastroesophageal Reflux Disease Among Surgical Patients During 1988-2012: A Single-institution, High-volume Experience in China. Annals of surgery.263(1):88-95.

4. Aburatani T, Kojima K, Otsuki S, Murase H, Okuno K, Gokita K, Tomii C, Tanioka T, Inokuchi M (2017) Double-tract reconstruction after laparoscopic proximal gastrectomy using detachable ENDO-PSD. Surgical endoscopy.31(11):4848-56.

5. Nakamura M, Nakamori M, Ojima T, Katsuda M, Iida T, Hayata K, Matsumura S, Kato T, Kitadani J, Iwahashi M, Yamaue H (2014) Reconstruction after proximal gastrectomy for early gastric cancer in the upper third of the stomach: an analysis of our 13-year experience. Surgery.156(1):57-63.

6. Writing committee of digestive tract reconstruction after proximal g (2020) [Chinese consensus on digestive tract reconstruction after proximal gastrectomy]. Zhonghua wei chang wai ke za zhi = Chinese journal of gastrointestinal surgery.23(2):101-8.

7. Aihara R, Mochiki E, Ohno T, Yanai M, Toyomasu Y, Ogata K, Ando H, Asao T, Kuwano H (2010) Laparoscopy-assisted proximal gastrectomy with gastric tube reconstruction for early gastric cancer. Surgical endoscopy.24(9):2343-8.

8. Kuroda S, Nishizaki M, Kikuchi S, Noma K, Tanabe S, Kagawa S, Shirakawa Y, Fujiwara T (2016) Double-Flap Technique as an Antireflux Procedure in Esophagogastrostomy after Proximal Gastrectomy. Journal of the American College of Surgeons.223(2):e7-e13.

9. Wang Y, Li B, Shan F, Li S, Xue K, Miao R, Li Z (2023) A modified esophagogastric reconstruction method after laparoscopic proximal gastrectomy: A technical note with video vignette. Asian J Surg.

10. (2021) Japanese gastric cancer treatment guidelines 2018 (5th edition). Gastric cancer : official journal of the International Gastric Cancer Association and the Japanese Gastric Cancer Association.24(1):1-21.

11. Clavien PA, Barkun J, de Oliveira ML, Vauthey JN, Dindo D, Schulick RD, de Santibañes E, Pekolj J, Slankamenac K, Bassi C, Graf R, Vonlanthen R, Padbury R, Cameron JL, Makuuchi M (2009) The Clavien-Dindo classification of surgical complications: five-year experience. Annals of surgery.250(2):187-96.
