# Supplementary material for: Safety and short-term outcomes of a modified valvuloplastic esophagogastrostomy versus gastric tube anastomosis after laparoscopy-assisted proximal gastrectomy: a retrospective cohort study
Source: Surg Endosc. 2024 Jan 25;38(3):1523–32. doi: 10.1007/s00464-023-10663-0 (PMC10881610; doi:10.1007/s00464-023-10663-0)
Supplement: Supplementary file 3 — Supplementary file3 (DOCX 20 kb) [file 464_2023_10663_MOESM3_ESM.docx]

Supplementary Table 1. Demographic and short-term outcomes of patients receiving endoscopy and not receiving endoscopy in the arch-bridge anastomosis group.

| Clinical parameters | Receiving endoscopy (n=13) | Not receiving endoscopy (n=12) | P value |
| --- | --- | --- | --- |
| Age (years) | 64.5±10.2 | 59.6±7.9 | 0.170 |
| Sex |  |  | 1.000 |
| Male | 11(84.6) | 10(83.3) |  |
| Female | 2 (15.4) | 2 (16.7) |  |
| ECOG PS |  |  | 0.302 |
| 0 | 6(46.2) | 8(66.7) |  |
| 1 | 7(53.8) | 4(33.3) |  |
| BMI (kg/m^2^) | 25.0±3.4 | 24.9±3.2 | 0.950 |
| Tumor location |  |  | 0.543 |
| EGJ | 7(53.8) | 5(41.7) |  |
| U | 6(46.2) | 7(58.3) |  |
| Tumor size (cm) | 2.5(1.7,3.5) | 2.0(1.9,2.8) | 0.852 |
| Pathological stage  IA  IB | 6(46.1)  4(30.8) | 4(33.3)  5(41.7) | 0.728 |
| IIA  IIB | 0(0.0)  0(0.0) | 1(8.3)  0(0.0) |  |
| IIIA  Operation time  Postoperative complications  Yes  No  Use of PPI | 3(23.1)  188.8±31.8  2(15.4)  11 (84.6)  0 (0) | 2(16.7)  180.7±16.7  2(16.7)  10 (83.3)  0(0) | 0.479  1.000  1.000 |

*ECOG PS* Eastern cooperation Oncology Group performance status; *BMI* body mass index; *EGJ* esophagogastric junction; *U* upper third; *PPI* proton pump inhibitor

Supplementary Table2. Demographic and short-term outcomes of patients receiving endoscopy and not receiving endoscopy in the gastric tube anastomosis group.

| Clinical parameters | Receiving endoscopy (n=8) | Not receiving endoscopy (n=10) | P value |
| --- | --- | --- | --- |
| Age (years) | 57.9±5.1 | 61.4±7.8 | 0.290 |
| Sex |  |  | 0.559 |
| Male | 6(75.0) | 9(90.0) |  |
| Female | 2(25.0) | 1(10.0) |  |
| ECOG PS |  |  | 0.367 |
| 0 | 6(75.0) | 5(50.0) |  |
| 1 | 2(25.0) | 5(50.0) |  |
| BMI (kg/m^2^) | 24.5(23.0,27.8) | 23.0(21.8,25.3) | 0.274 |
| Tumor location |  |  | 0.638 |
| EGJ | 6(75.0) | 6(60.0) |  |
| U | 2(25.0) | 4(40.0) |  |
| Tumor size (cm) | 2.5±1.4 | 3.0±0.9 | 0.375 |
| Pathological stage  IA  IB | 4(50.0)  1(12.5) | 3(30.0)  0(0.0) | 0.633 |
| IIA  IIB | 1(12.5)  0(0.0) | 5(50.0)  2(20.0) |  |
| IIIA  Operation time  Postoperative complications  Yes  No  Use of PPI  Yes  No | 2(25.0)  211.0(192.8,234.8)  3 (37.5)  5 (52.5)  3(37.5)  5(62.5) | 0(0.0)  219.5(188.8,272.5)  3 (30.0)  7 (70.0)  1(10.0)  9 (90.0) | 0.829  1.000  0.275 |

*ECOG PS* Eastern cooperation Oncology Group performance status; *BMI* body mass index; *EGJ* esophagogastric junction; *U* upper third; *PPI* proton pump inhibitor
